# Scalable Fabrication of Methylammonium-Free Wide-Bandgap Perovskite Solar Cells by Blade Coating in Ambient Air

**DOI:** 10.1007/s40820-025-01838-6

**Published:** 2025-07-01

**Authors:** Jianbo Liu, Meng Zhang, Xiaoran Sun, Linhu Xiang, Xiangyu Yang, Xin Hu, Zhicheng Wang, Tian Hou, Jinzhao Qin, Yuelong Huang, Mojtaba Abdi-Jalebi, Xiaojing Hao

**Affiliations:** 1 School of New Energy and Materials, Southwest Petroleum University, Chengdu, 610500 People’s Republic of China; 2 The Australian Centre for Advanced Photovoltaics, School of Photovoltaic and Renewable Energy Engineering, University of New South Wales, Sydney, NSW 2052 Australia; 3 Huzhou Phoenixolar Co., Ltd., Huzhou, 313000 People’s Republic of China; 4 Institute for Materials Discovery, University College London, Malet Place, London, WC1E 7JE UK

**Keywords:** Blade coating, MA-free perovskite ink, Wide-bandgap perovskite, Perovskite solar cells, Large area

## Abstract

Scalable fabrication of efficient wide-bandgap (WBG) perovskite solar cells (PSCs) is crucial to realize the full commercial potential of tandem solar cells. However, there are challenges in fabricating efficient methylammonium-free (MA-free) WBG PSCs by blade coating, especially its phase separation and films stability. In this work, an MA-free WBG perovskite ink is developed for preparing FA_0.8_Cs_0.2_Pb(I_0.75_Br_0.25_)_3_ films by blade coating in ambient air. Among various A-site iodides, RbI is found to be the most effective in suppressing the precipitation of PbI_2_ induced by Pb(SCN)_2_ while keeping the enlarged grains. The distribution of Rb suggested that the Rb ions are kept isolated with the perovskite grains during the crystallization and Ostwald ripening processes, which contributes to the formation of the large-grain WBG perovskite film with minimum non-radiative recombination. As a result, a power conversion efficiency (PCE) of 23.0% was achieved on small-area WBG PSCs, while mini-modules with an aperture area of 10.5 cm^2^ exhibited a PCE of 20.2%, among the highest reported for solar cells prepared with WBG perovskites via blade coating. This work presents a scalable and reproducible fabrication strategy for stable MA-free WBG PSCs under ambient conditions, advancing their path toward commercialization.

**Supplementary Information:**

The online version contains supplementary material available at 10.1007/s40820-025-01838-6.

## Highlights

- RbI is the most effective in mitigate PbI_2_ precipitation caused by Pb(SCN)_2_ while maintaining large grains.
- Rb is kept at the grain boundaries during crystallization and Ostwald ripening, contributes to a slow growth of the grains.
- Wide-bandgap perovskite solar cells with blade-coated perovskite in air achieved a certified power conversion efficiency of 23%, among the highest values reported.

**Supplementary Information:**

The online version contains supplementary material available at 10.1007/s40820-025-01838-6.

## Graphical abstract

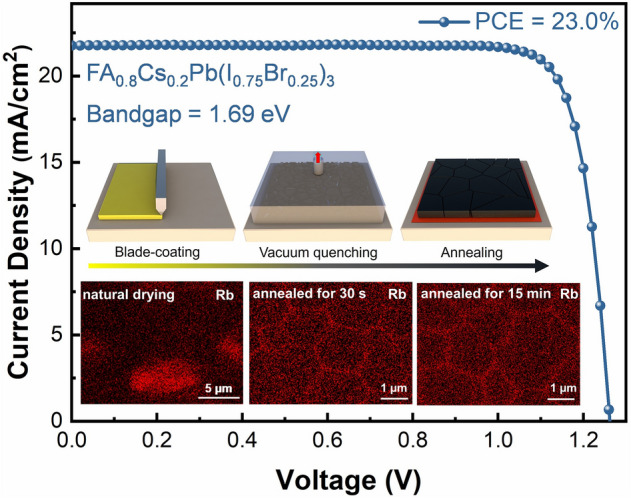

## Introduction

Over the past decade, organic–inorganic hybrid perovskite materials have garnered significant attention in the photovoltaics (PV) application due to their outstanding optoelectronic properties and robust solution processable fabrication [1–7]. Because of its tunable bandgaps, perovskite can be integrated with many traditional PV materials for tandem cells, yielding higher power conversion efficiency (PCE) than single junction solar cells [8–11]. Recently, the perovskite/silicon tandem cells employing wide-bandgap (WBG) (~ 1.7 eV) perovskites demonstrate certified PCE over 34%, showing its remarkable potential for next generation PV technology [12]. However, the fabrication of WBG perovskite mostly relies on spin-coating, which is not favorable for large-scale production of WBG perovskite films [13, 14]. Developing scalable manufacturing of WBG perovskite films remains crucial to their practical application.

Blade coating is one of the most promising methods to prepare large-area thin films because of its high material efficiency and ease of operation [15, 16]. Significant advancements have been made in advancing these coating technologies in the recent years. For instance, Seo et al. utilized blade coating methods to fabricate n-i-p perovskite solar cells (PSCs) with a 1.65 eV bandgap, achieving impressive PCEs of 20.56% for devices with an active area of 0.094 cm^2^ and 18.4% for those measuring 1 cm^2^ [17]. Fang et al. reported PCEs of 22.06% and 19.63% for blade-coated p-i-n configuration PSCs with a 1.67 eV bandgap, covering active areas of 0.07 and 1.02 cm^2^, respectively [18]. More recently, Wolf et al. attained a notable PCE of 22.6% with blade coating for devices featuring a 1.66 eV bandgap and an active area of 0.1 cm^2^, highlighting their great potential for tandem PV applications [19]. However, these WBG perovskite components typically contain methylammonium (MA) cations, which dissociate easily under light, leading to additional proton migration and raising concerns about the long-term stability of these devices [20–23]. Moreover, perovskite undergoes degradation during preparation in ambient air [24–26], thus requiring the processing in inert atmosphere or controlled humidity of less than 25% [27]. Otherwise, the degradation causes MA^+^ to deprotonate, forming MA^0^, which reacts with FAI to produce MFAI as a byproduct [28, 29]. To overcome these challenges in ambient processing, our recent work on pure-phase FAPbI_3_ highlights the importance of constricting stable intermediate phase with 2-imidazolidinone (IMD), which could be also promising for blade coating of WBG perovskite thin films in air [30].

To obtain high crystallinity of WBG perovskite, additive engineering using Pb(SCN)_2_ has been demonstrated essential to enhance crystallization and mitigate phase separation in WBG perovskites [10, 31]. However, employing Pb(SCN)_2_ additives comes at a significant cost of precipitating excess PbI_2_. Although the surface PbI_2_ can be reduced by surface passivation [32], PbI_2_ persists in the bulk and can be decomposed into metallic Pb^0^ and I_2_ under light exposure [33]. Metallic Pb^0^ is considered an intrinsic factor that significantly impacts PSCs, leading to reduced device efficiency and stability [34]. Therefore, achieving high-quality WBG perovskite thin films while suppressing the precipitation of excess PbI_2_ is particularly critical to guarantee stable performance of the devices.

In this work, we introduced various A-site iodides into the MA-free WBG perovskite precursor solution to compensate the PbI_2_ precipitation induced by Pb(SCN)_2_ in the crystallization stage_._ The PbI_2_ in the resulting MA-free WBG perovskite films can be eliminated by most of the A-site iodides. However, only RbI can retain the enlarged grains in the presence of Pb(SCN)_2_. This could be attributed to the possibility that the RbI does not interfere with the formation of WBG perovskite grains, as evidenced by the isolated distribution of Rb at the detected all stages of the crystallization process. The isolated Rb distribution with respect to Pb during both natural drying and different crystallization stages implies limited contribution of Rb in forming the bulk wide-bandgap perovskite grains. The incorporation of Pb(SCN)_2_ and RbI promotes grain fusion, resulting in a compact and large-grain morphology of the blade-coated perovskite films with reduced non-radiative recombination losses. The corresponding devices fabricated in ambient air achieved PCEs of 23.0% for 0.093 cm^2^ devices and 20.2% for 10.5 cm^2^ mini-modules. These efficiencies represent some of the highest reported for MA-free WBG PSCs prepared by blade coating.

## Experimental Section

### Materials

Dimethylformamide (DMF), aluminum oxide (Al_2_O_3_), and isopropanol (IPA) were procured from Sigma Aldrich. Ethylenediamine dihydroiodide (EDAI_2_) and (4-(7H-dibenzo[c,g]carbazol-7-yl)butyl) phosphonic acid (4PADCB) was acquired from LiWei Materials. Formamidinium iodide (FAI), Caesium iodide (CsI, 99%), Lead (II) iodide (PbI_2_), Lead bromide (PbBr_2_), and Phenylethylammonium iodide (PEAI) was obtained from Xi’an Elante New Material Co., Ltd. 2-Imidazolidinone (IMD), Rubidium iodide (RbI), and Lead sulfocyanide (Pb(SCN)_2_) were purchased from Aladdin. Fullerene (C_60_) was acquired from Xi’an Yuri Solar Co., Ltd. All chemicals were utilized as received without subsequent purification.

### Device Fabrication

#### Preparation of Perovskite Precursor Solution

To prepare the FA_0.8_Cs_0.2_Pb(I_0.75_Br_0.25_)_3_ precursor solution, a mixture of FAI, CsI, PbI_2_, PbBr_2_, and IMD (in a molar ratio of 80:20:75:25:20) was dissolved in DMF to a concentration of 1.5 M and shaken at room temperature for 30 min until completely dissolved (IMD is a solid Lewis base used as an additive). For the Pb(SCN)_2_-treated precursor solution, 1.5% (relative to the molar amount of Pb) of Pb(SCN)_2_ was added. In the target precursor solution, 1.5% Pb(SCN)_2_ and 3% RbI (with 3% FAI or 3% CsI) were added.

#### Perovskite Solar Cell Fabrication

The ITO conductive glass substrate was cleaned and ultrasonically treated for 20 min using ionized water (containing 5% detergent by volume), deionized water, and ethanol, respectively, dried with dry air, and then treated with ultraviolet ozone for 15 min. 4PADCB (0.5 mg mL^−1^, dissolved in ethanol) was deposited on the ITO substrate at 3000 r min^−1^ for 30 s and then annealed at 100 °C for 10 min. The Al_2_O_3_ dispersion solution (with a dilution ratio of 1:50 of the stock solution to IPA) was spin-coated on the 4PADCB film at 5000 r min^−1^ for 30 s and heated at 100 °C for 10 min. Then, the PEAI solution (1 mg mL^−1^, dissolved in DMF) was spin-coated at 5000 r min^−1^ for 30 s and then kept at 100 °C on a hot plate for 5 min. The process of preparing the perovskite film in air is as follows: the perovskite precursor solution (usually 15 µL for a 2.5 × 2.5 cm^2^ substrate and 30 µL for a 5 × 5 cm^2^ substrate) is dropped into the gap between the scraper and the substrate (~ 150 µm), then scraped at a speed of 5 mm s^−1^. The wet film is then quickly transferred to a vacuum chamber for a vacuum flash process. During this process, the vacuum flash time is evacuated to about 10 Pa within 20 s, and the film is then annealed on a hot stage at different temperatures for 20 min. PEAI and EDAI_2_ were dissolved in IPA (1 mg mL^−1^ each). The solution was ultrasonicated for 1 h to ensure complete dissolution and filtered through a 0.22 µm organic membrane filter before use. After the perovskite layer was prepared, the mixed solution of PEAI and EDAI_2_ was spin-coated onto the top of the perovskite film at 5000 r min^−1^ for 30 s and then kept on a hot plate at 100 °C for 10 min. A 20 nm layer of C_60_ was continuously thermally evaporated at an evaporation rate of 0.5 Å s^−1^ under a pressure of about 1 × 10^–3^ Pa. Subsequently, 20 nm of SnO_2_ was deposited on top of the C_60_ substrate in an ALD reactor at 100 °C via 75 cycles. Each ALD cycle consisted of an 8 s TDMA dose, followed by an 18 s purge, a 6 s water vapor dose, and another 18 s purge. Silver (120 nm) was thermally evaporated under vacuum to form the electrode. Finally, a 100 nm layer of LiF was thermally evaporated at a rate of 1 Å s^−1^ as an antireflection layer.

Mini-module Fabrication: Mini-modules were fabricated using the same p-i-n device structure as employed in small-area cells. The process began with laser etching of the P1 pattern onto the ITO glass substrate using a 1064 nm wavelength laser. Subsequently, following the ALD of the SnO_2_ layer, the P2 pattern was defined using a 532 nm laser. Gold electrode deposition was then performed, after which the P3 pattern was etched with the 532 nm laser. Finally, a 1064 nm laser was utilized for edge cleaning to complete the module fabrication. This sequential laser patterning process achieved a mini-module with an effective active area of 10.5 cm^2^.

### Characterization of Devices and Films

The X-ray diffraction (XRD) analysis of the film series was performed using an X-ray diffractometer (Dandong Tongda Science and Technology Co., Ltd.) with Cu Kα radiation (λ = 1.5418 Å). Surface morphology of the films was examined via top-view scanning electron microscopy (SEM) using a KYKY-EM8000 microscope (Beijing KYKY Optic-electronics Technology Co., Ltd.). Elemental composition was analyzed through energy-dispersive X-ray spectroscopy (EDS) on a Regulus 8100 instrument (HITACHI). Ultraviolet–visible (UV–vis) absorption spectra were recorded using a Q6 spectrometer (Shanghai Metash Instruments Co., Ltd.). Dynamic light scattering (DLS) measurements were conducted on a Malvern Zetasizer Nano ZS90. Steady-state photoluminescence (PL) and time-resolved photoluminescence (TRPL) spectra were obtained using an Edinburgh Instruments FLS 980 spectrometer, equipped with a 468 nm continuous wave excitation source and a 406 nm pulsed laser, respectively. The current–voltage (*J-V*) characteristics of perovskite solar cells were measured with a Keithley B2901A source meter under simulated AM 1.5 G illumination (100 mW cm^−2^) provided by an Enli Tech solar simulator. External quantum efficiency (EQE) measurements were conducted under ambient conditions using an EnliTech EQE system. Monochromatic light, chopped at a frequency of 210 Hz, was used to illuminate the devices, and their photocurrent was measured using a lock-in amplifier. Atomic force microscopy (AFM) and Kelvin probe force microscopy (KPFM) imaging were performed with a KEYSIGHT Technologies 7500 AFM. Ultraviolet photoelectron spectroscopy (UPS) was carried out on an ESCALAB XI + (Thermo Fisher Scientific).

## Results and Discussion

### Preparation of WBG Perovskite Films via Blade Coating

**Fig. 1:**
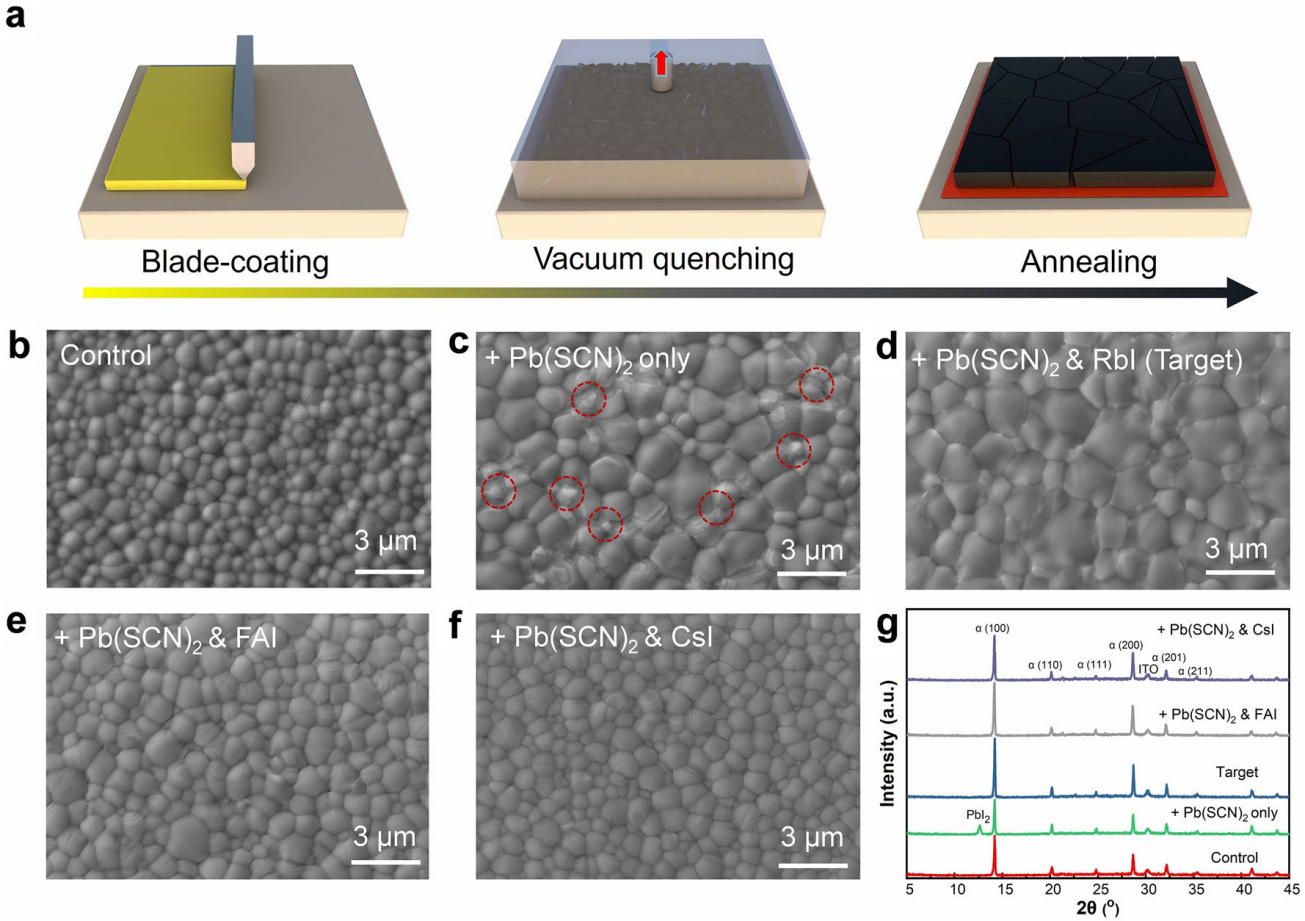
**a** Schematic diagram of the blade coating vacuum-assisted preparation of FA_0.8_Cs_0.2_Pb(I_0.75_Br_0.25_)_3_ perovskite films. Top-view SEM images of perovskite films of **b** control, **c** Pb(SCN)_2_-only, **d** Pb(SCN)_2_ and RbI, **e** Pb(SCN)_2_ and FAI, and **f** Pb(SCN)_2_ and CsI. **g** XRD patterns of the blade-coated WBG perovskite films

Fig. 1a presents a schematic diagram illustrating the preparation of MA-free WBG perovskite films via blade coating in ambient air. The blade-coated wet film initially appears transparent, then turns brown upon vacuum quenching, and becomes nearly opaque after thermal annealing (Fig. S1). The WBG perovskite studied in this work has a composition of FA_0.8_Cs_0.2_Pb(I_0.75_Br_0.25_)_3_ as determined by its precursor. Its bandgap of ~ 1.7 eV lies in the optimal bandgap range for perovskite/c-Si tandem solar cells. By using IMD to control the intermediate phase [30], a multicrystalline MA-free WBG perovskite films can be prepared as noted as control sample (Figs. 1b and S2). However, its grain size is much smaller than that of narrow bandgap perovskite [30], which can be ascribed to the rapid crystallization of the Br-rich phase due to its lower solubility and fast ion diffusion [35, 36] and thereby not favorable in maximizing the PV performance of the device. In contrast, the incorporation of Pb(SCN)_2_ effectively enlarged the grain size of the films. Unfortunately, the stoichiometric mismatch (i.e. extra Pb) led to the precipitation of PbI_2_ as indicated in Fig. 1c, g. In order to balance the stoichiometry in the precursors to mitigate the PbI_2_ formation caused by Pb(SCN)_2_, various A-site iodides were introduced into the precursor solution. It is found that RbI, FAI, and CsI can all effectively inhibit the precipitation of PbI_2_ (Fig. 1d-g). However, the addition of FAI and CsI, while eliminating PbI_2_, also reduces the perovskite grain size close to that of the control sample. Contrastingly, only RbI added sample (noted as target) retained the large-grain size of the MA-free WBG perovskite. According to the comparison of XRD patterns and SEM images (Fig. S3), 3% RbI is identified as the optimal concentration to achieve phase-pure perovskite without secondary byproducts (e.g., RbPbI_3_). During the natural drying process (Fig. S4), the persistence duration of intermediate phase diffraction peaks (6.1°, 8.9°) in target films exhibits increase compared to the control, indicating suppressed nucleation kinetics and prolonged crystallization timeframe. This delayed phase evolution process is conducive to the formation of perovskite films with larger grain sizes.

### Growth and Photophysical Characterization of WBG Perovskite Films

**Fig. 2:**
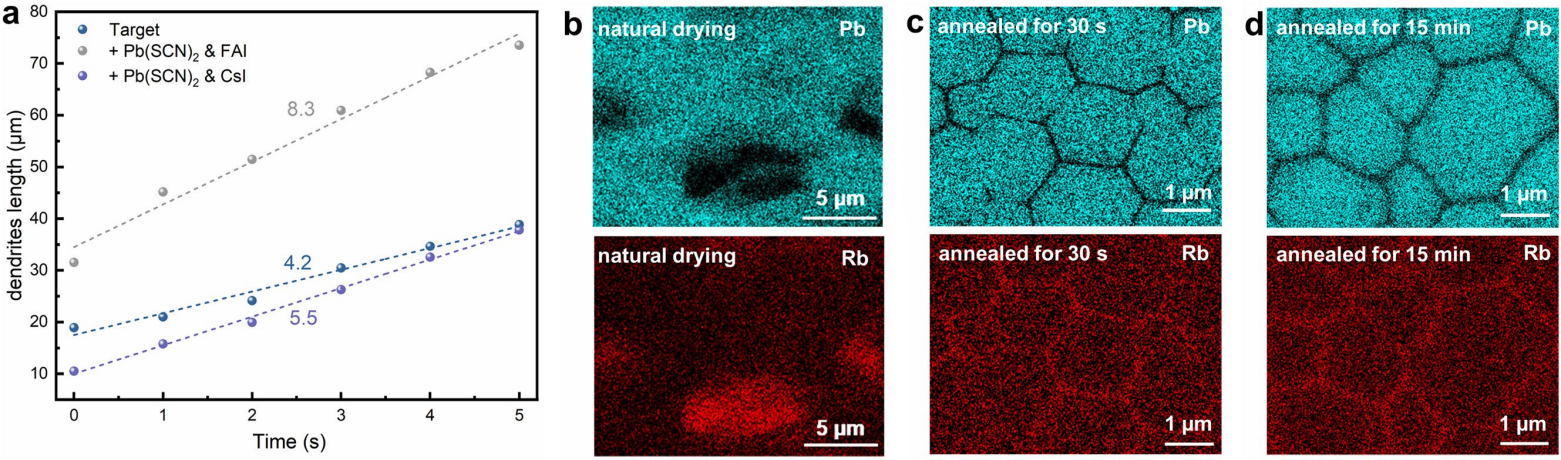
**a** Line graph of dendrite length change during natural drying of WBG precursor films. EDS mapping of target films of Pb and Rb elements under different conditions: **b** natural drying, **c** annealed for 30 s, and **d** annealed for 15 min

Given the markedly different chemical environment of the IMD-based precursor solutions compared to conventional precursor systems [37–40], the natural drying process of these precursor solutions are firstly investigated to understand the unique role of RbI with other A-site iodides. By monitoring the dendritic growth under optical microscope (Figs. 2a and S5), it is found the target group with RbI shows slowest nuclei growth rate of 4.2 µm s^−1^, in contrast to 8.3 and 5.5 µm s^−1^ of the FAI group and CsI group, respectively. The slow nuclei growth indicates the nucleation and crystal growth of the target group are suppressed by the presence of Rb cation, which could be potentially account for the larger grain size observed in the annealed film. The addition of RbI inhibits colloidal aggregation, yielding smaller particle sizes (Fig. S6). This size reduction elevates the nucleation energy barrier, which may correlate with the delayed nucleation observed during natural drying. Furthermore, the elemental distribution of the films at different stages are acquired by EDS mapping. Fig. 2b-d shows the distribution of Pb and Rb in the target films crystalized under natural drying, annealing for 30 s, and 15 min, respectively. The natural dried film shows large dendrite morphology, while the annealed films show multicrystalline morphology (Fig. S7). The increased grain size from annealed 30 s to 15 min can be ascribed to an Ostwald ripening growth. It can be observed that Rb are always distributed opposite to Pb (Fig. 2) and other elements (Fig. S8), while distributed uniformly along the vertical profile of the perovskite layer (Fig. S9). After natural drying Rb are found at the gaps between the dendrites rather than on the dendrites. And in both Fig. 2c, d, Rb is mostly found enriched at the grain boundaries of the perovskite film, suggesting Rb is highly likely not participate the growth (construction and merge) of the perovskite grains, and forced to diffuse into the final grain boundaries during the crystalline growth. Compared to FA and Cs, which can be directly consumed in the local grain growth, the diffusion of Rb would require longer time and therefore results in slow growth rate and large grains. Both RbI and RbCl additives yield perovskite films with suppressed PbI_2_ formation and kept enlarged grains, confirming the critical role of Rb⁺ in crystallization kinetics (Fig. S10). The presence of Rb is also identified by XPS (Fig. S11) and XRD (Fig. S12), indicating that Rb exists in the form of RbPbI_3,_ which suggests the reaction occurs when mixing Pb(SCN)_2_ and RbI (Fig. S13).

**Fig. 3:**
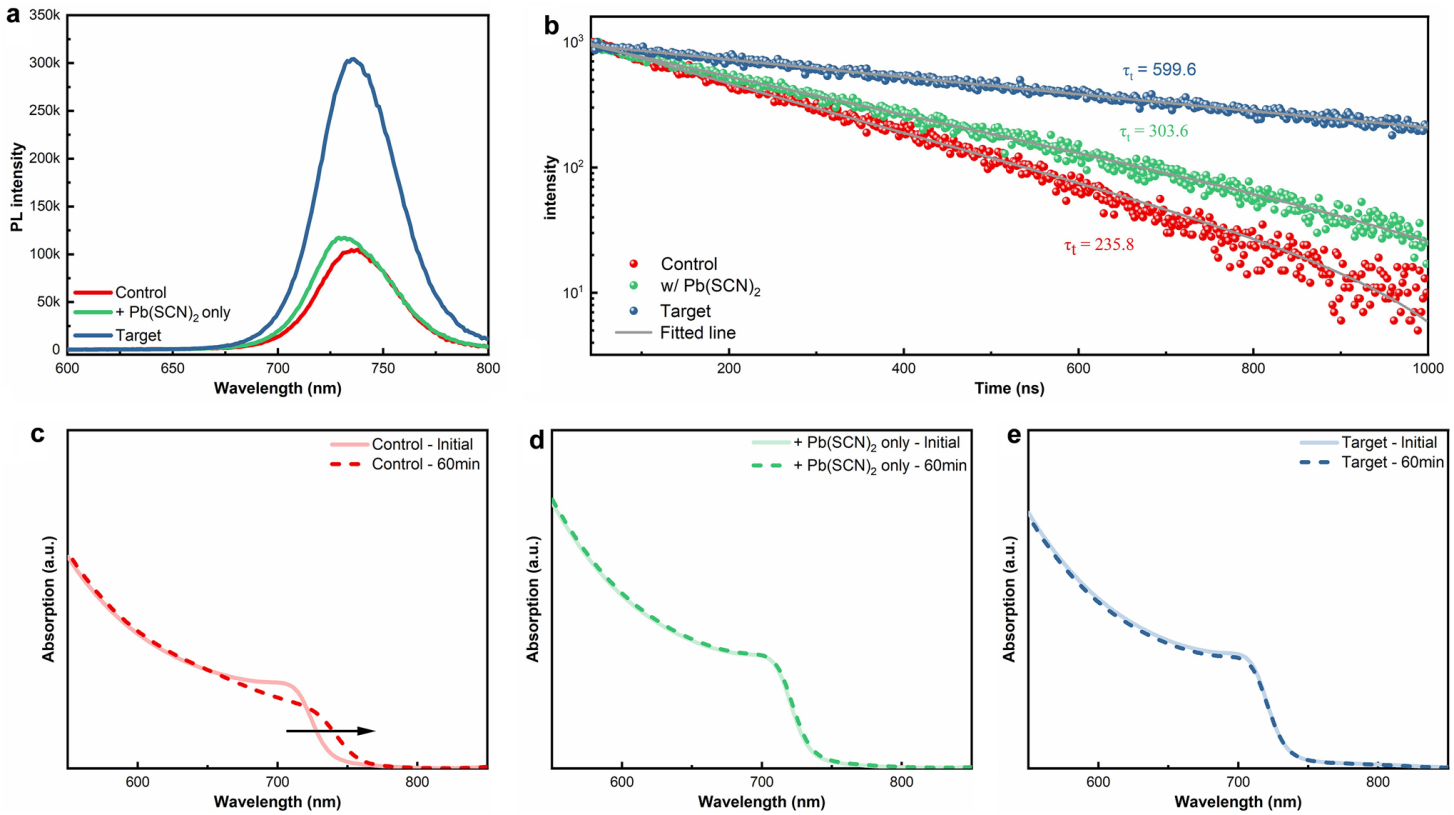
**a** PL and **b** TRPL spectra of control, Pb(SCN)_2_-only, and target perovskite films. UV–Vis absorption spectra of **c** control, **d** Pb(SCN)_2_-only, and **e** target perovskite films before and after 60 min 1-sun intensity illumination

The incorporation of RbI in combination with Pb(SCN)_2_ not only eliminated the precipitation of PbI_2_ but also improved the crystallinity of MA-free WBG perovskite film. This can be evidenced by the narrow full width at half maximum (FWHM) of the (100) diffraction peak of the target sample compared to the control sample and Pb(SCN)_2_-only sample (Fig. S14). The best annealing temperature is estimated to be 120 °C for the target sample evidenced by the narrowest FWHM of the diffraction peak (Fig. S15). It is speculated that the rapid removal of the IMD ligand at higher temperature could disrupt the fine growth of the target film grains. PL and TRPL measurements were employed to study the defects of the blade-coated MA-free WBG perovskite films. As shown in Fig. 3a, the target films exhibited significantly higher PL intensity compared to the control and Pb(SCN)_2_-only films, which can be attributed to its improved crystallinity. Meanwhile, the TRPL decay curves in Fig. 3b were analyzed to estimate the carrier lifetimes of the films. The addition of Pb(SCN)_2_ has improved the carrier lifetimes from 235.8 to 303.7 ns, which can be further improved to 599.6 ns by RbI addition (target). The more effectively suppressed non-radiative recombination indicates reduced defect density in the target film compared to the control and Pb(SCN)_2_-only films.

Subsequently, the phase-stability of the blade-coated MA-free WBG perovskite film was evaluated by light-soaking the sample films under 1 sun illumination for 60 min (Fig. 3c-e). The control film exhibits significant changes at its absorption onset, indicating bandgap changes due to the photo-induced halide segregation [41]. In contrast, the Pb(SCN)_2_-only and target films showed negligible changes before and after the light soaking. The demonstrated decent phase-stability of target film under illumination is considered very beneficial in enhancing the operational stability of the WBG perovskite solar cells.

### Energy Band Analysis of WBG Perovskite Films

**Fig. 4:**
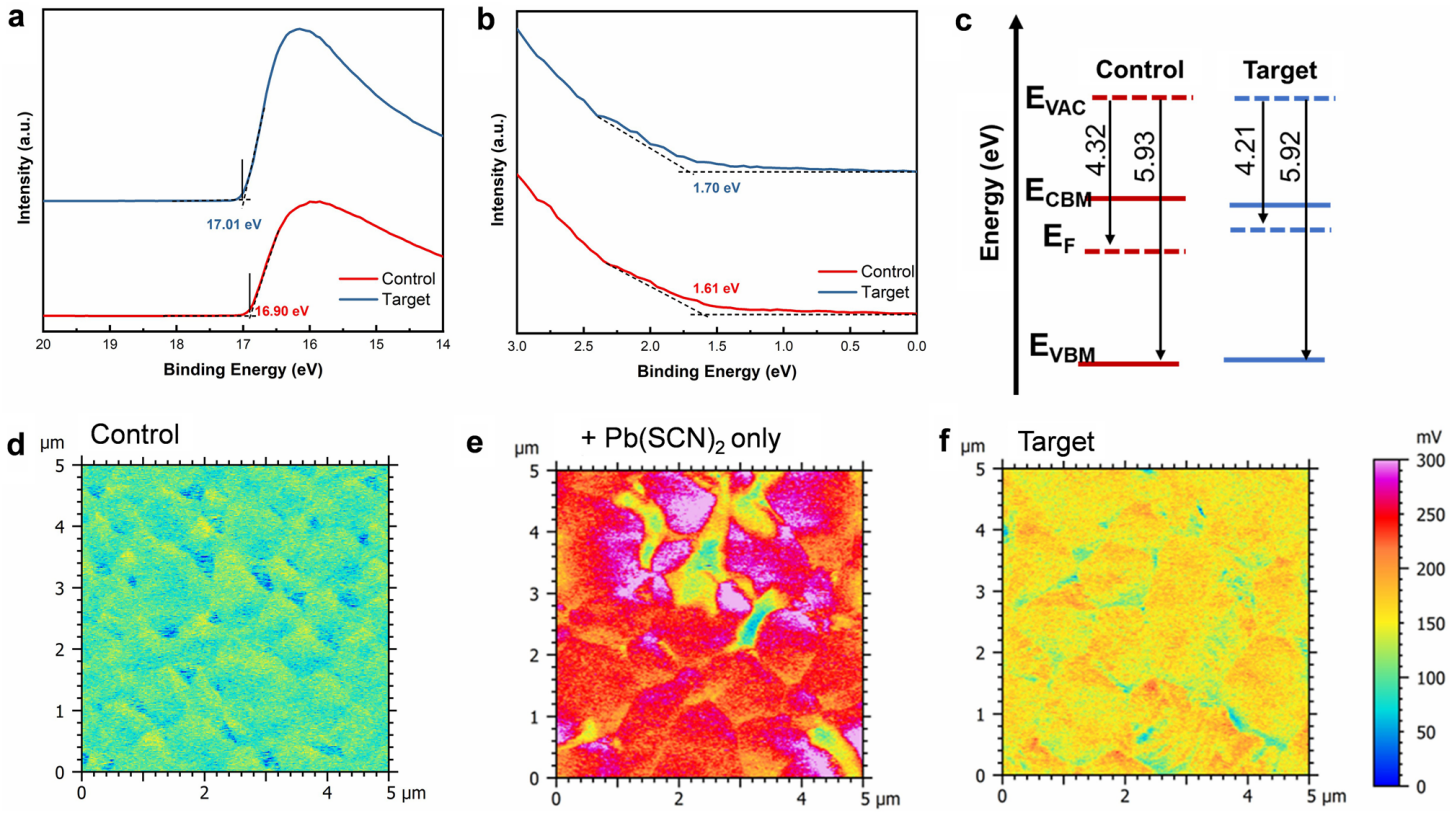
**a** Secondary electron cut-off spectrum of the control and target perovskite films. **b** The valence band spectra of the control and target perovskite films. **c** Energy level diagram of control and target perovskite films. Contact potential difference images of the **d** control, **e** Pb(SCN)_2_-only, and **f** target perovskite films by KPFM

Furthermore, the energy levels including conduction band minimum, Fermi level (*E*_*F*_), and valence band maximum of the blade-coated MA-free WBG perovskite films were investigated by analyzing the UPS (Fig. 4a, b) and UV–vis absorption spectra (Fig. S16). The work function measurement reveals a notable increase from 4.32 eV for the control perovskite film to 4.21 eV for the target perovskite film, representing an upward shift of 0.11 eV (Fig. 4c) [42]. To further investigate the surface potential of the perovskite films, KPFM measurements were conducted under ambient conditions. The target film exhibits a lower and more uniform surface potential compared to the Pb(SCN)_2_-only films, but a higher surface potential relative to the control film (Figs. 4 d-f and S17). This trend is consistent with the *E*_*F*_ results obtained from UPS measurements. Specifically, the *E*_*F*_ of the target perovskite film shifts closer to the conduction band, which contributes to an increase in the open-circuit voltage (*V*_*OC*_) [43].

### PV Performance and Stability of WBG PSCs

**Fig. 5:**
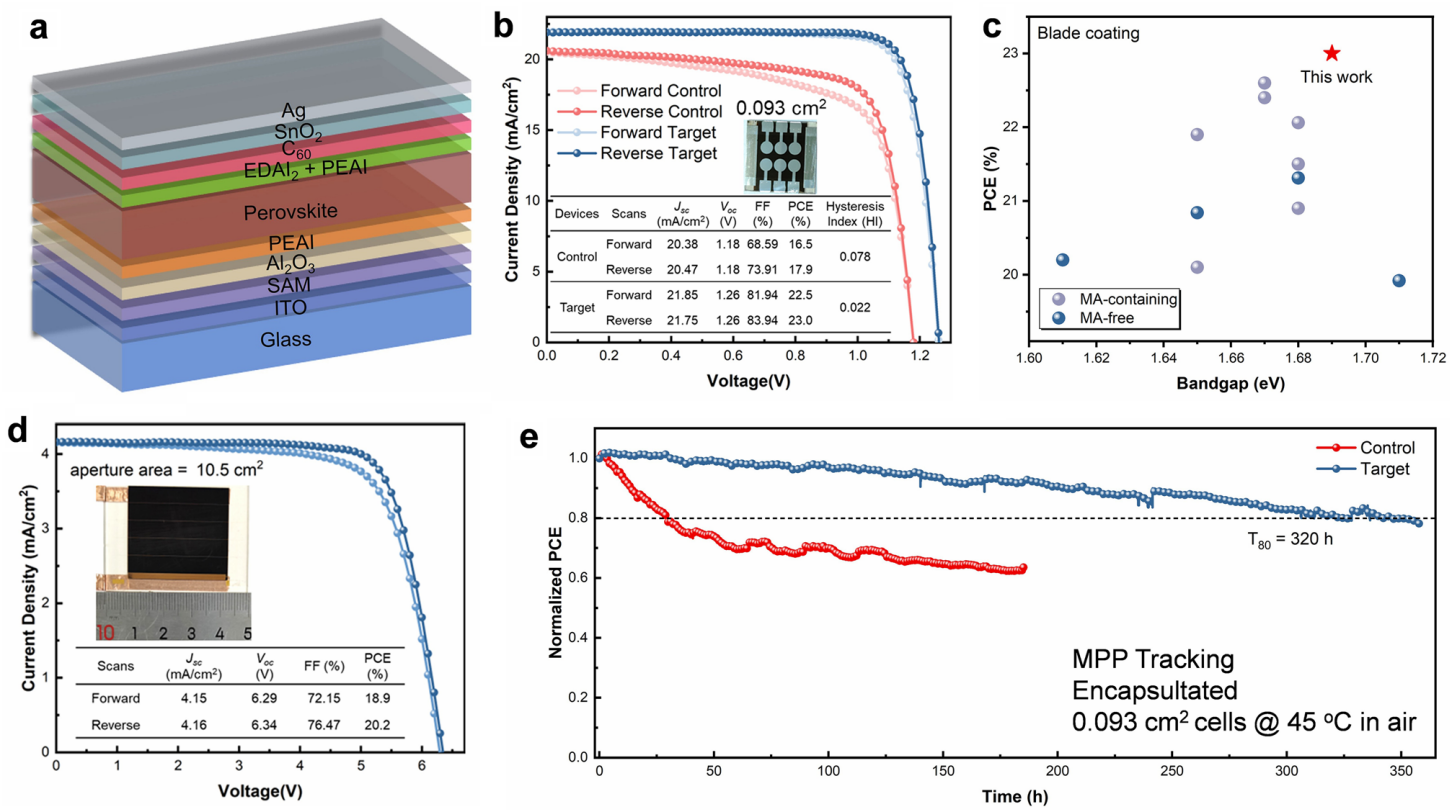
**a** Device architecture of the inverted WBG PSCs. **b** *J-V* curves of the control and target devices, and corresponding photos. **c** Champion PCEs of WBG PSCs fabricated by blade coating from MA-free and MA-containing inks as a function of the perovskite films. **d** Best *J-V* curve of the mini-modules (aperture area: 10.5 cm^2^). **e** Maximum power point tracking of control and target devices in an ambient atmosphere (45 °C)

To evaluate the device performance of the blade-coated MA-free WBG perovskite film, PSCs with a structure ITO/4PADCB/Al_2_O_3_/PEAI/FA_0.8_Cs_0.2_Pb(I_0.75_Br_0.25_)_3_/PEAI + EDAI_2_/C_60_/SnO_2_/Ag were fabricated (Fig. 5a) [30]. The cross-sectional SEM image of the corresponding target device is shown in Fig. S18. The best *J-V* curve is presented in Fig. 5b, where the champion device achieved a PCE of 23.0%, representing one of the highest values reported for WBG PSCs with bandgaps ranging from 1.65 to 1.71 eV, as shown in Fig. 5c and Table S1. This device exhibited negligible hysteresis, a high *V*_*OC*_ of 1.26 V, a short-circuit current density (*J*_*SC*_) of 21.75 mA cm^−2^, and an impressive fill factor (FF) of 83.94%. In comparison, the control device showed inferior photovoltaic performance, with a PCE of 17.9%, *V*_*OC*_ of 1.18 V, *J*_*SC*_ of 20.47 mA cm^−2^, and FF of 73.91%. The improved *Voc* of the target device is also demonstrated by an enlarged built-in potential from the Mott-Schottky measurements (Fig. S19a). And the impedance spectroscopy and dark current measurements further confirm the suppression of non-radiative recombination in the target device (Fig. S19b, c), which is in a good agreement with the presented result of the film characterization. The EQE spectra of the devices (Fig. S20) corroborate the enhancement in *J*_*SC*_ of the target device, consistent with the *J-V* curve. A certified PCE of 23.0% was also obtained from a third-party organization (Fig. S21), To assess reproducibility, statistical data from forward and reverse scans of 30 devices were collected (Fig. S22), showing a narrow performance distribution for the target devices. To assess the scalability of the WBG perovskite ink, XRD and SEM analyses were conducted at five distinct regions of the blade-coated 5 × 5 cm^2^ film. The data reveal uniform crystallographic properties and morphological homogeneity, as shown in Fig. S23, validating the ink’s suitability for large-area fabrication. Subsquently, mini-modules with 10.5 cm^2^ aperture area (geometric fill factor is 0.985, Fig. S24) are fabricated with the same p-i-n device structure to small-area cells to further demonstrate the upscaling feasibility. The champion mini-modules achieved a reverse scan PCE of 20.2% with *J*_*SC*_ of 4.16 mA cm^−2^, *V*_*OC*_ of 6.34 V, and FF of 76.47% as shown in Fig. 5d. The mini-modules exhibit more pronounced J-V hysteresis compared to small-area cells, likely due to unoptimized interfaces introduced during the scaling-up process. This leads to a faster PCE decay in MPPT measurements, which, however, can be fully recovered after resting in the dark (Fig. S25). Additionally, the WBG perovskite films demonstrated excellent phase stability, showing no significant changes after heating at 65 °C for 60 min under one-sun irradiation. (Fig. S26). Compared to the control device, the target device exhibited markedly superior operational stability (Fig. 5e), retaining 80% of its initial PCE after 320 h of maximum power point (MPP) tracking under continuous illumination at approximately 45 °C. As shown in Fig. S27, the target devices retained 92% of their initial PCE after 1,100 h of storage under ambient conditions (30 ± 10% RH, 25 °C), showing improved stability over the control devices, which preserved merely 73% of their initial PCE.

## Conclusions

In summary, a MA-free WBG perovskite ink was successfully developed for the fabrication of FA_0.8_Cs_0.2_Pb(I_0.75_Br_0.25_)_3_ films using blade coating under ambient air conditions. To address PbI_2_ precipitation induced by Pb(SCN)_2_, various A-site iodides were introduced into the perovskite precursor solution. Among them, RbI proved to be the most effective additive in suppressing PbI_2_ precipitation while maintaining large-grain sizes. The spatial distribution analysis of Rb ions indicated that they remain segregated from the perovskite grains during crystallization and Ostwald ripening, which plays a critical role in enabling the formation of large-grain WBG perovskite films with suppressed non-radiative recombination. Consequently, small-area WBG PSCs achieved a PCE of 23.0%, while mini-modules with a 10.5 cm^2^ aperture attained a PCE of 20.2%, among the highest values reported for blade-coated WBG PSCs. These results highlight the potential of scalable MA-free WBG PSCs fabrication as a simple, reproducible, and efficient approach for producing high-performance photovoltaic devices under ambient air conditions.

## Supplementary Information

Below is the link to the electronic supplementary material.

Supplementary file1 (DOCX 3513 KB)
