# Supplementary material for: Scalable Fabrication of Methylammonium-Free Wide-Bandgap Perovskite Solar Cells by Blade Coating in Ambient Air
Source: Nanomicro Lett. 2025 Jul 1;17:318. doi: 10.1007/s40820-025-01838-6 (PMC12214146; doi:10.1007/s40820-025-01838-6)
Supplement: Supplementary file 1 — Supplementary file1 (DOCX 3513 KB) [file 40820_2025_1838_MOESM1_ESM.docx]

Supporting Information for

**Scalable Fabrication of Methylammonium-Free Wide-Bandgap Perovskite Solar Cells by** **Blade Coating in Ambient Air**

Jianbo Liu^1^, Meng Zhang^2,^ *, Xiaoran Sun^1^, Linhu Xiang^1^, Xiangyu Yang^1^, Xin Hu^3^, Zhicheng Wang^1^, Tian Hou^1^, Jinzhao Qin^3^, Yuelong Huang^1^^, 3,^ *, Mojtaba Abdi-Jalebi^4,^ *, and Xiaojing Hao^2,^ *

^1^ School of New Energy and Materials, Southwest Petroleum University, Chengdu 610500, P. R. China

^2^ The Australian Centre for Advanced Photovoltaics, School of Photovoltaic and Renewable Energy Engineering, University of New South Wales, Sydney, New South Wales 2052, Australia

^3^ Huzhou Phoenixolar Co., Ltd., Huzhou 313000, P. R. China

^4^ Institute for Materials Discovery, University College London, Malet Place, London WC1E 7JE, UK

# *Corresponding authors. E-mail: [meng.zhang@unsw.edu.au](mailto:meng.zhang@unsw.edu.au) (Meng Zhang); [hyl@phoenixolar.com](mailto:hyl@phoenixolar.com) (Yuelong Huang); [m.jalebi@ucl.ac.uk](mailto:m.jalebi@ucl.ac.uk) (Mojtaba Abdi-Jalebi); [xj.hao@unsw.edu.au](mailto:xj.hao@unsw.edu.au) (Xiaojing Hao)

# S1 Supplementary Figures and Tables


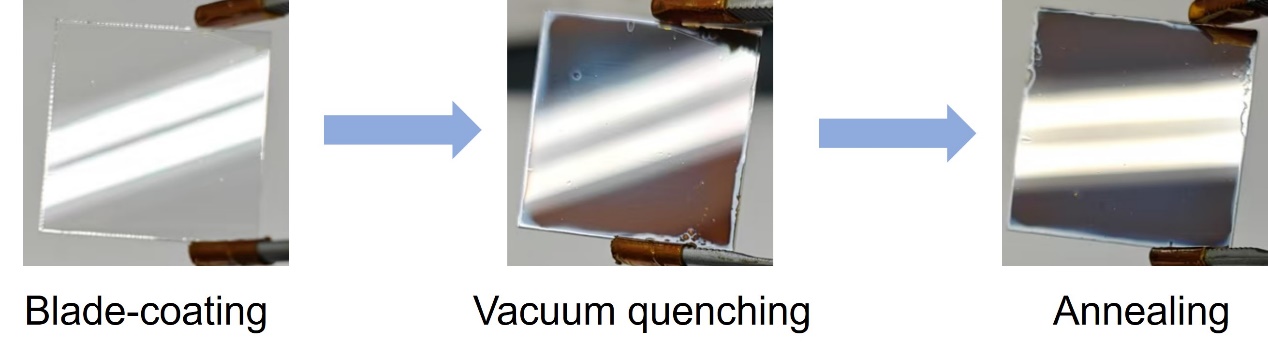


**Fig. S1** Photos of blade-coated WBG perovskite films at different stage


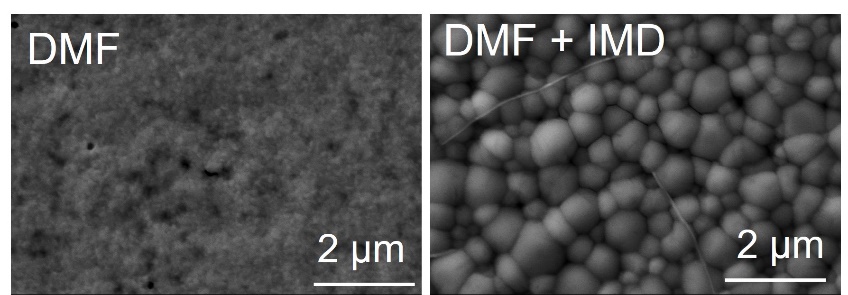


**Fig. S2** SEM images of blade-coated WBG perovskite films fabricated using DMF and DMF + IMD solvent systems


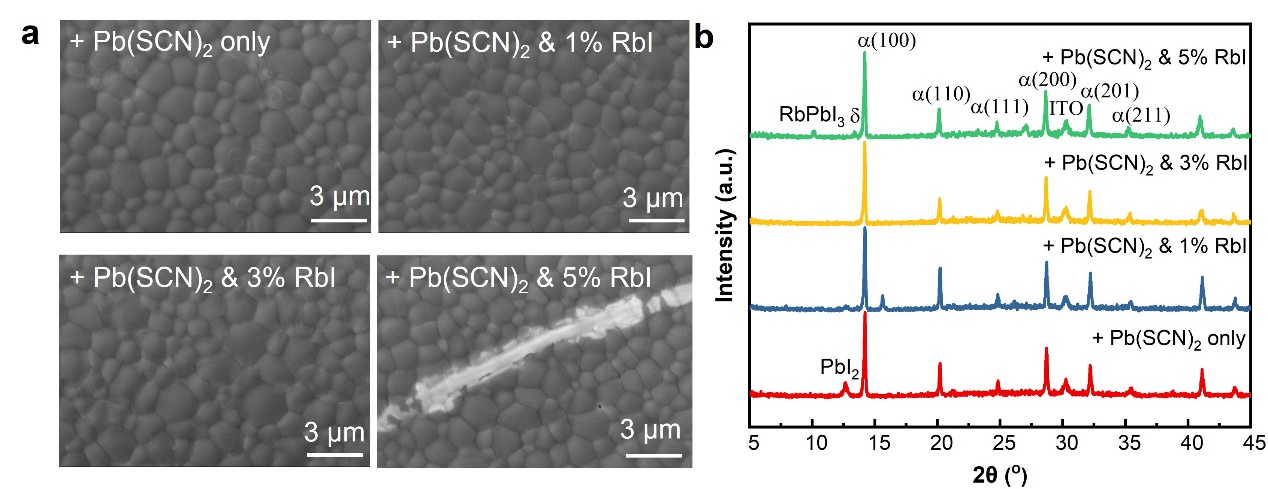


**Fig. S3** **a** SEM images and **b** XRD patterns of perovskite films with varying RbI concentrations


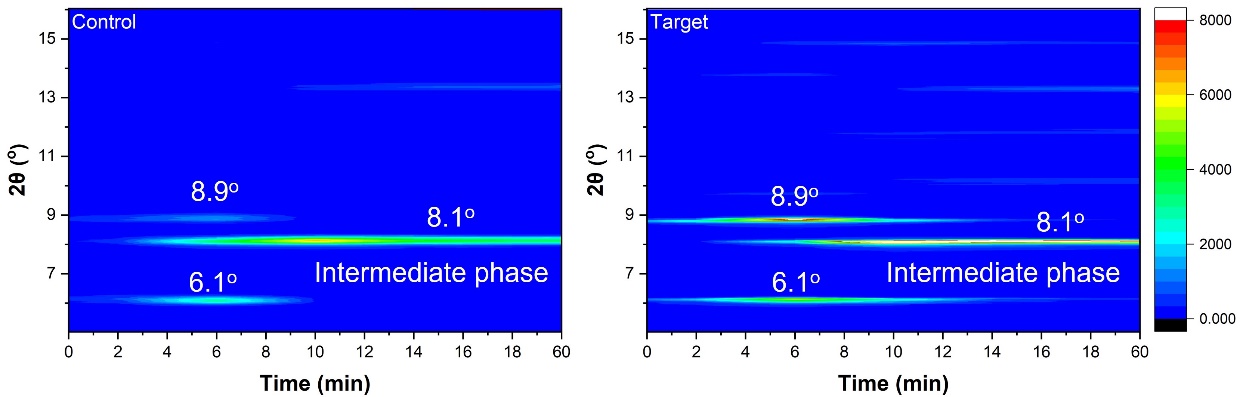


**Fig. S4** Time-resolved XRD analysis of phase evolution in control and target perovskite films during ambient drying


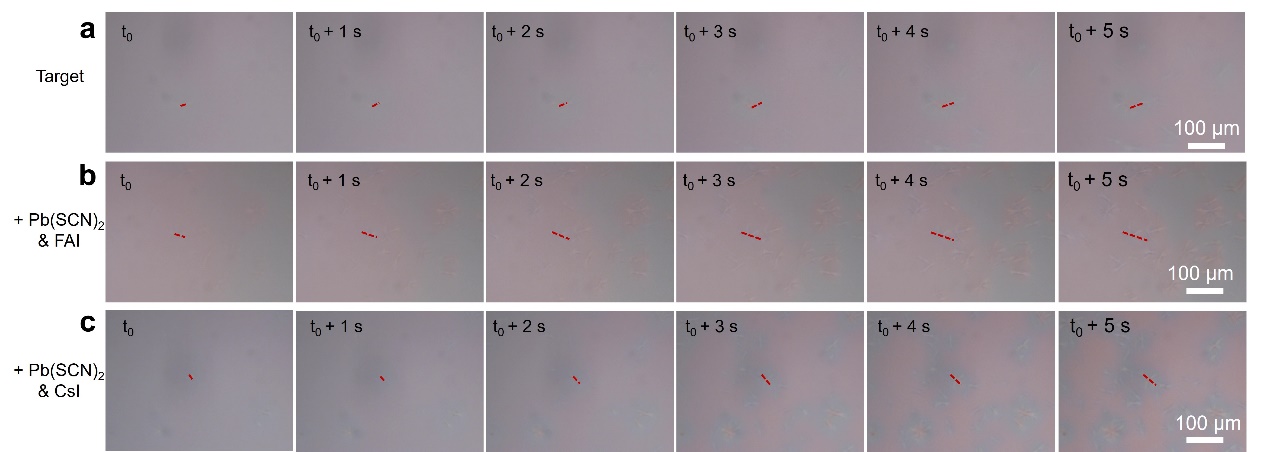


**Fig. S5** The dendrite growth during natural drying of WBG precursor films with **a** RbI, **b** FAI, and **c** CsI


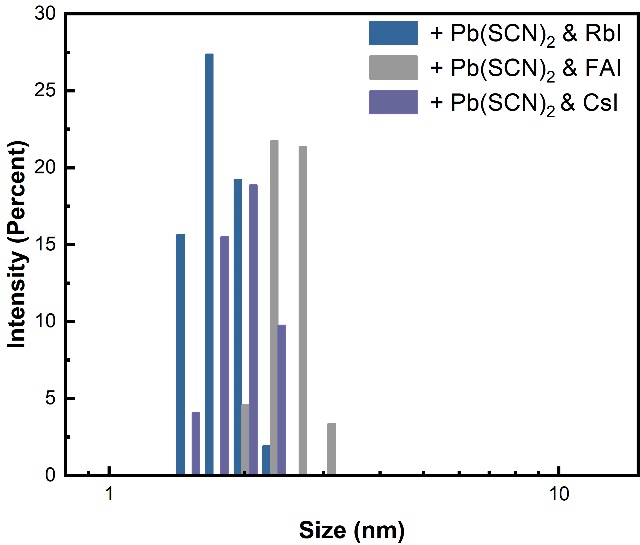


**Fig. S6** Dynamic light scattering (DLS) size distributions of perovskite precursor solutions with RbI, FAI, and CsI (test concentration: 1.5 M)


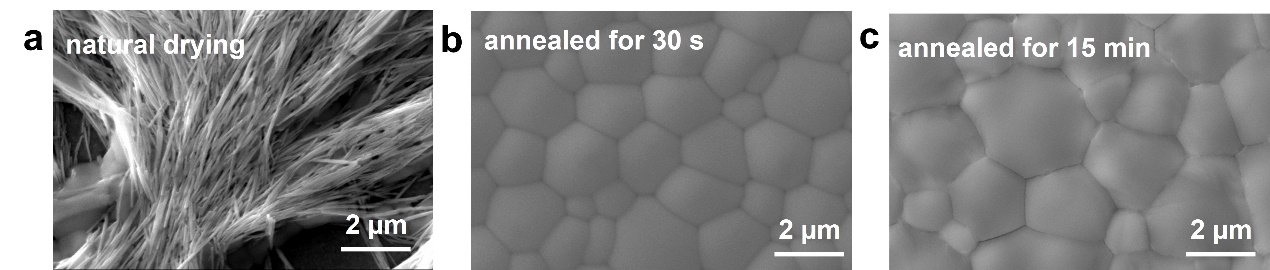


**Fig. S7** SEM images of target films under different conditions: **a** natural drying, **b** annealed for 30 s, and **c** annealed for 15 min


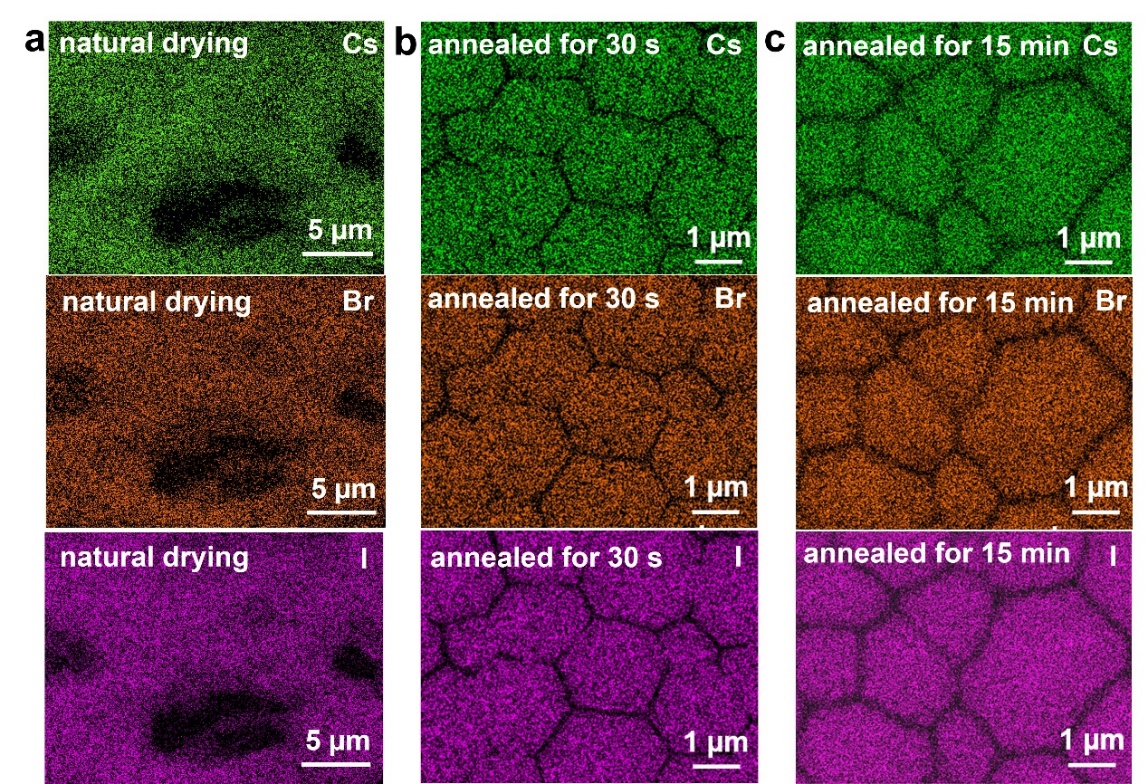


**Fig. S8** EDS mapping of target films of Cs, Br, and I elements under different conditions: **a** natural drying, **b** annealed for 30 s, and **c** annealed for 15 min


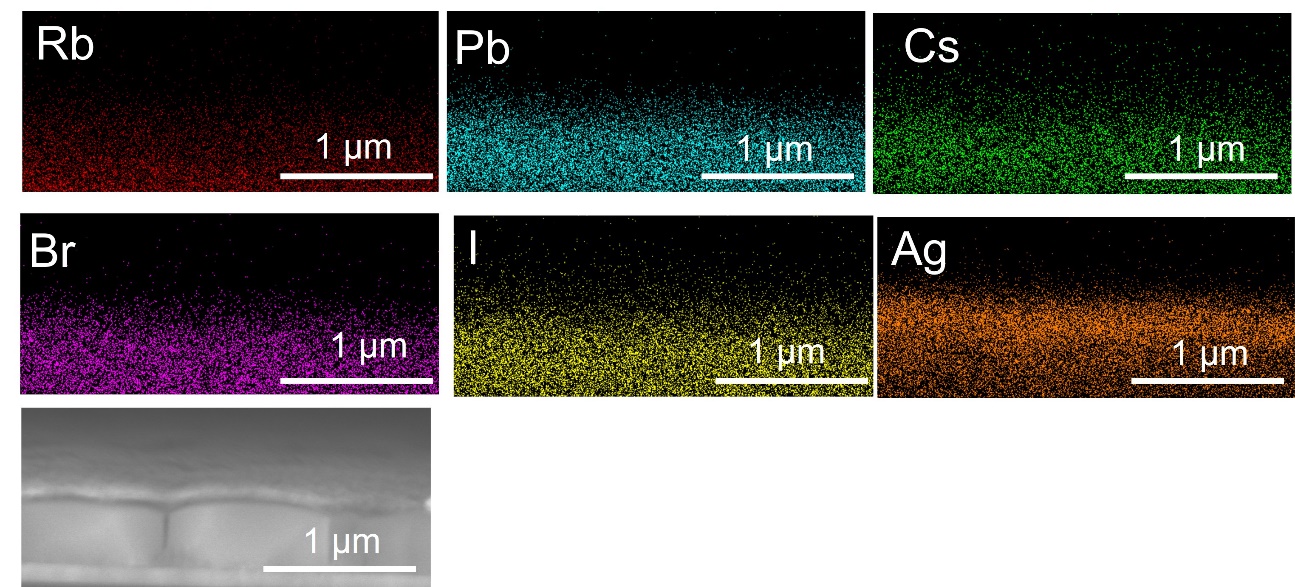


**Fig. S9** Cross-sectional EDS elemental mapping of the target perovskite film


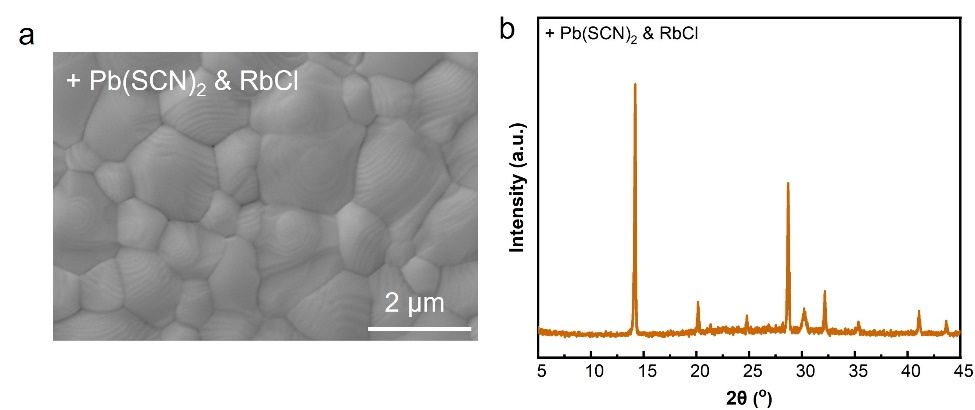


**Fig. S10 a** SEM image and **b** XRD pattern of perovskite film with 3% RbCl


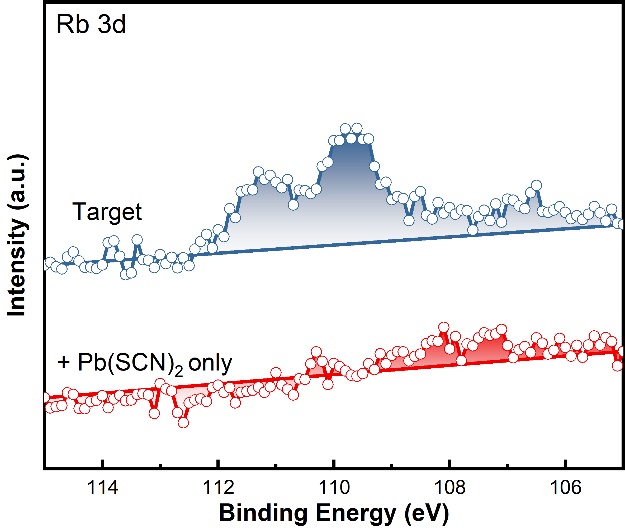


**Fig. S11** XPS spectra of the Rb 3d regions

**
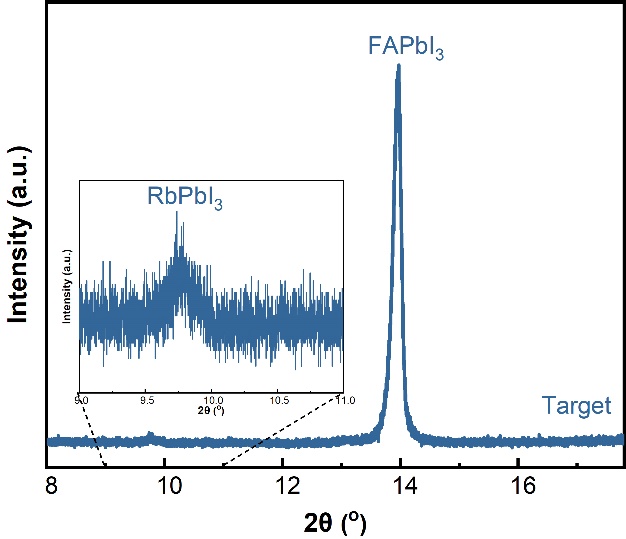
**

**Fig. S12** XRD pattern of target perovskite film

**
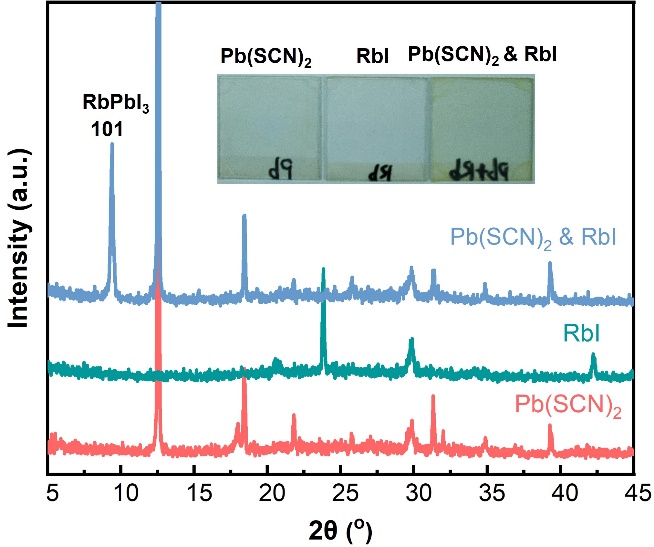
**

**Fig. S13** XRD patterns of Pb(SCN)_2_, RbI, and a mixture of Pb(SCN)_2_ and RbI (the inserted images are photos of the three films)


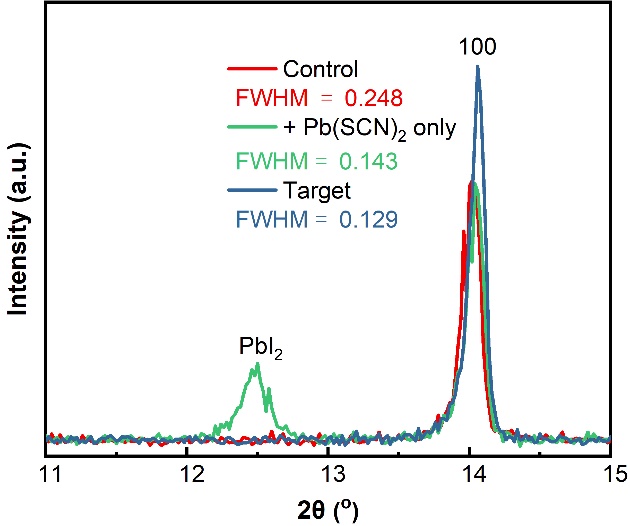


**Fig. S14** XRD patterns and FWHM of perovskite films


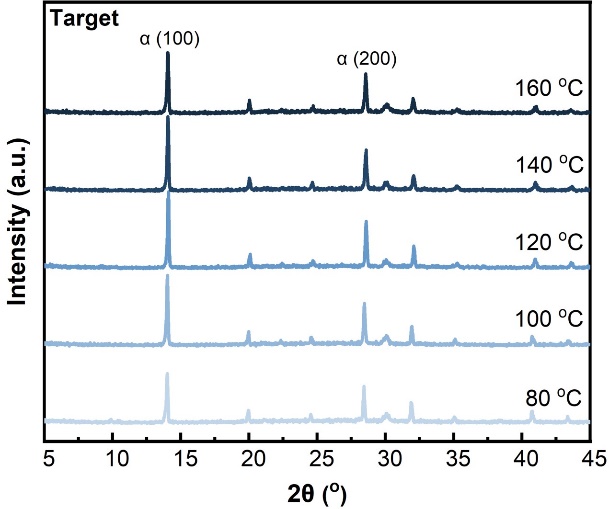


**Fig. S15** XRD patterns of the as-deposited perovskite precursor films before annealing

**
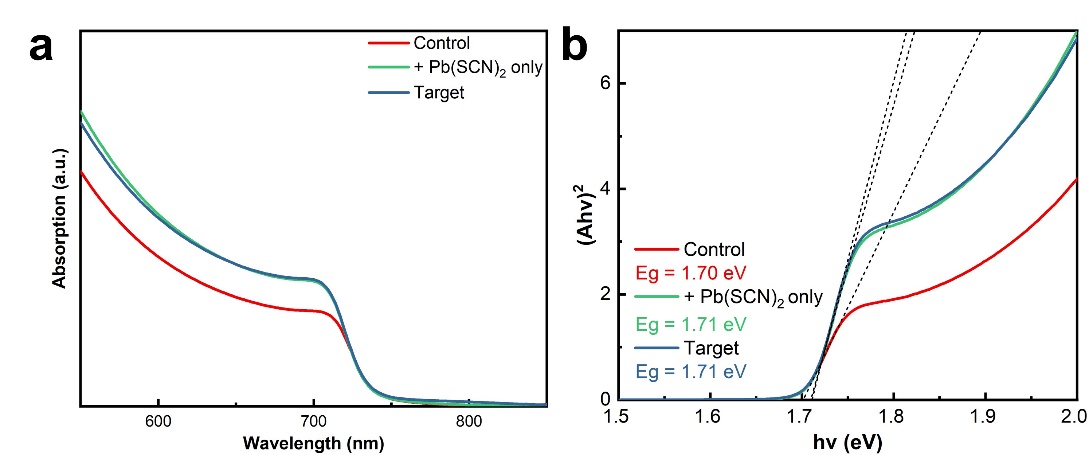
**

**Fig. S16 a** UV-vis absorption spectra and **b** Tauc plot of the FA_0.8_Cs_0.2_Pb(I_0.75_Br_0.25_)_3_ perovskite films

**
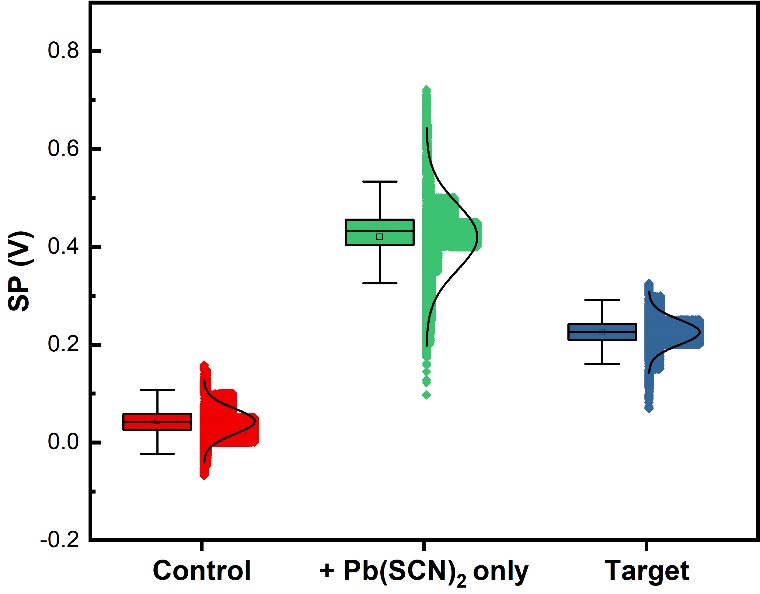
**

**Fig. S17** The surface potential (SP) statistics values from KPFM measurements for the control, Pb(SCN)_2_ only, and target films

**
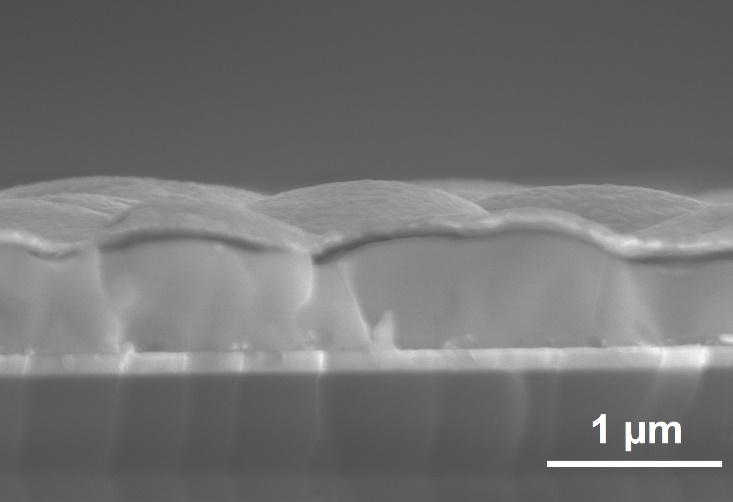
**

**Fig. S18** Cross-sectional SEM image of the target device


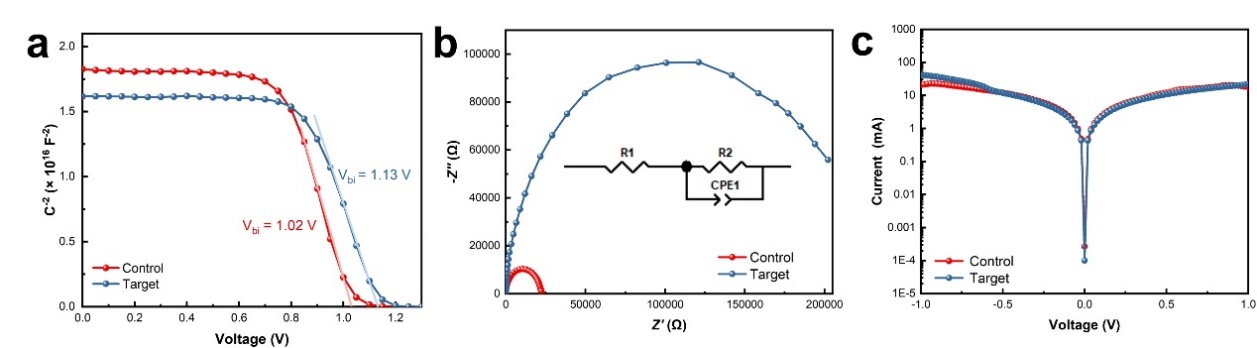


**Fig. S19 a** Mott-Schottky plots of the control and target devices; **b** Electrochemical impedance spectroscopy (EIS) spectrum of the control and target devices; **c** Dark *J-V* curves of the control and target devices


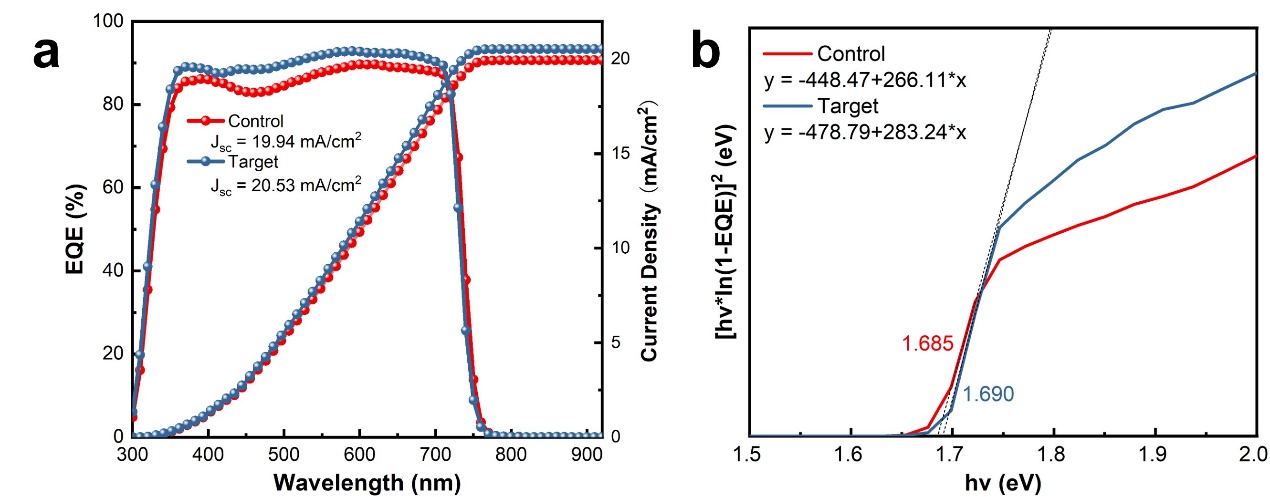


**Fig. S20 a** EQE spectra of control and target devices and **b** Bandgap values of control and target perovskite films, determined from EQE spectra


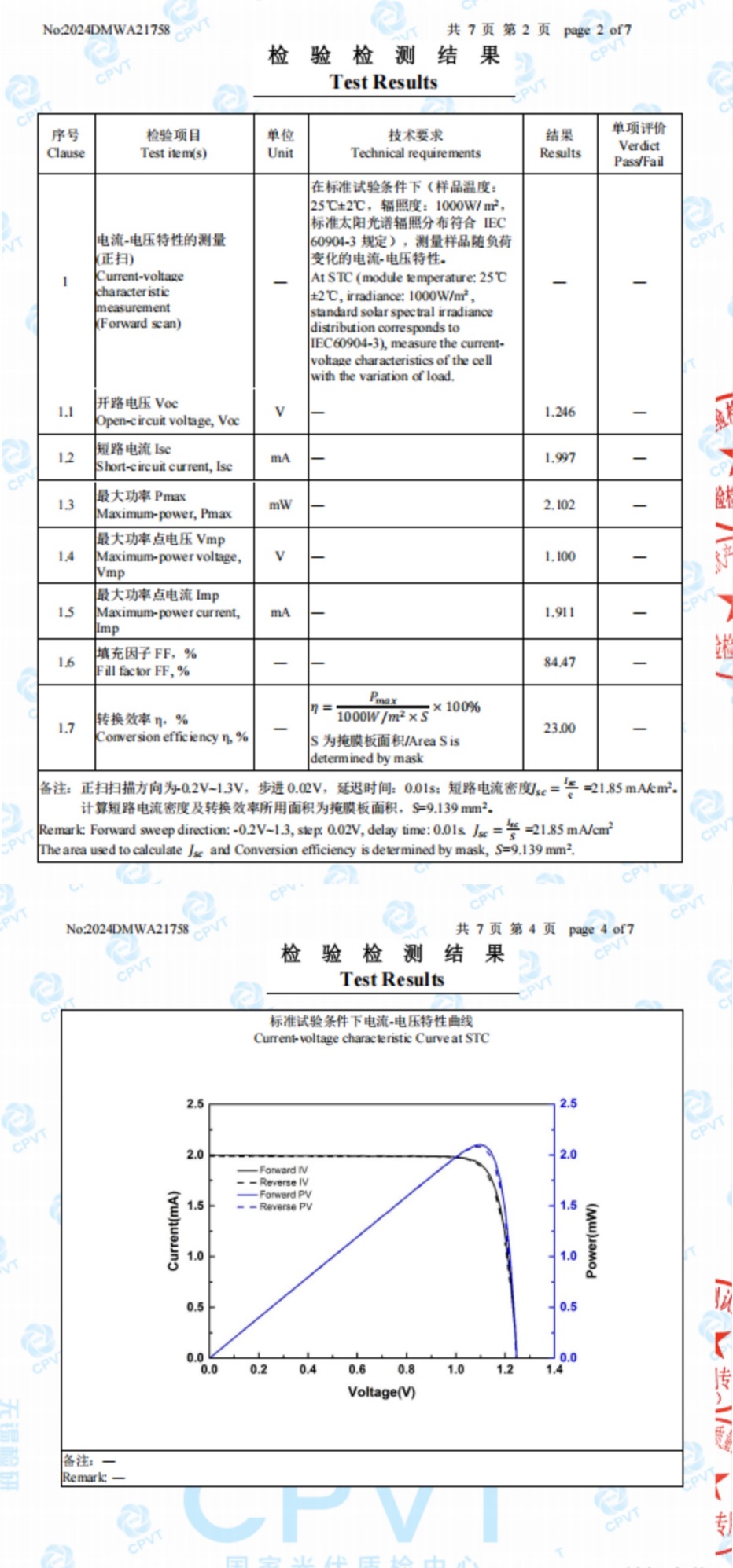


**Fig. S21** Certification report for small area WBG PSCs


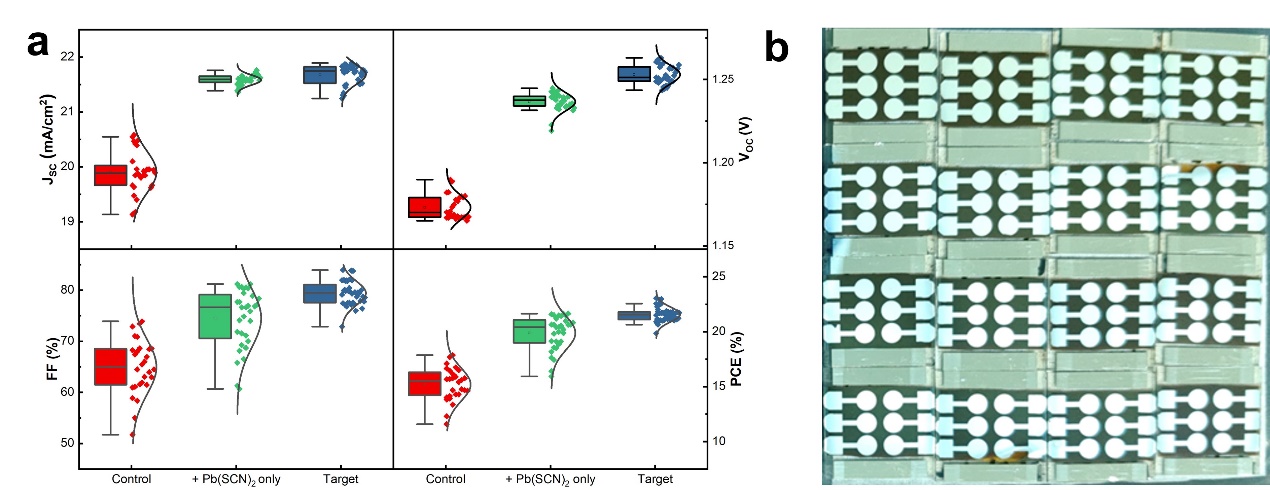


**Fig. S22 a** Distribution of PV parameters for the control, Pb(SCN)_2_ only, and target devices. **b** Photos of the devices


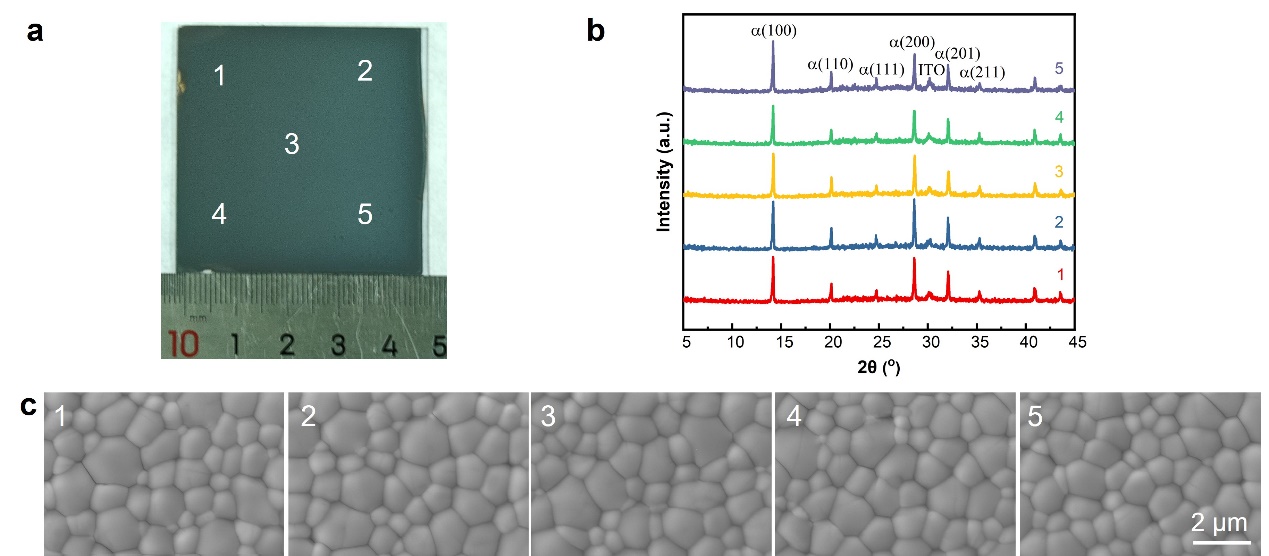


**Fig. S23** **a** Photograph of a 5×5 cm^2^ target film prepared by blade-coating. **b** XRD patterns and **c** SEM images collected at 5 different spots on the film


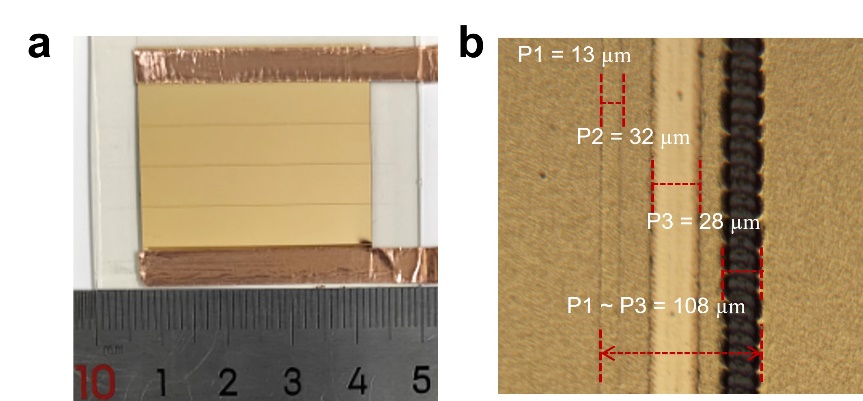


**Fig. S24** **a** Photos of the mini-module. **b** The optical image of the P1-P2-P3 patterning of the mini-module


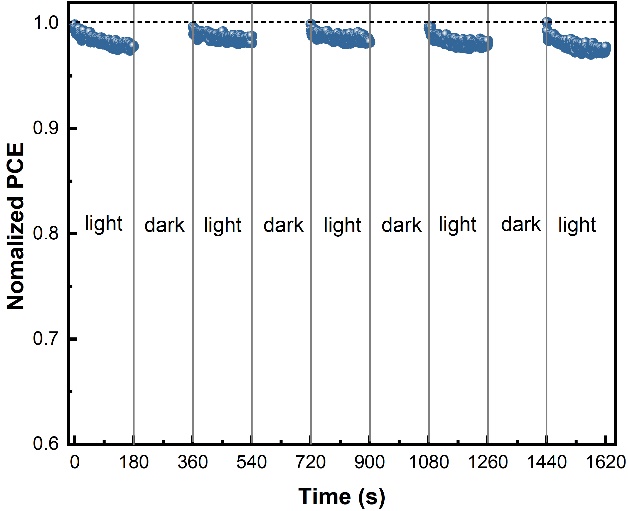


**Fig. S25** MPPT of the mini-module under multiple light/dark cycles

**
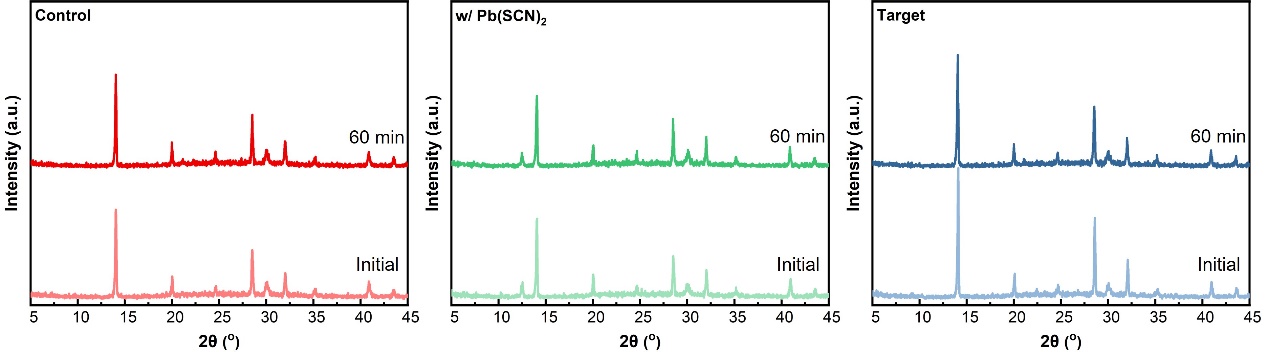
**

**Fig. S26** XRD patterns of FA_0.8_Cs_0.2_Pb(I_0.75_Br_0.25_)_3_ perovskite films before and after heating at 65 ^o^C for 60 minutes under one-sun irradiation


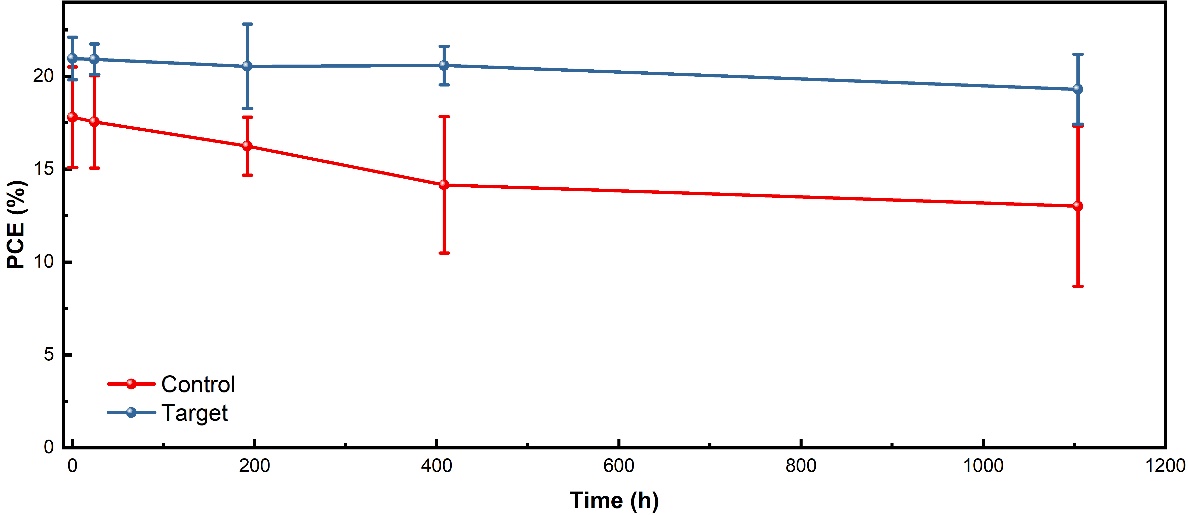


**Fig. S27** The storage stability of the control and target devices in air (at 25 °C with a relative humidity of 30 ± 10%)

**Table S1** Blade-coated champion single-junction WBG devices

| Bandgap (eV) | Component | Voc  (V) | Jsc  (mA/cm^2^) | FF  (%) | PCE  (%) | Area  (cm^2^) | Years |
| --- | --- | --- | --- | --- | --- | --- | --- |
| 1.65 | Cs_0.1_MA_0.9_Pb(I_0.9_Br_0.1_)_3_ | 1.167 | 21 | 82 | 20.1 | 0.06 | 2020 [S1] |
| 1.65 | Cs_0.1_FA_0.2_MA_0.7_Pb(I_0.85_Br_0.15_)_3_ | 1.23 | 21.2 | 83.8 | 21.9 | 0.06 | 2022 [S2] |
| 1.61 | FA_0.75_Cs_0.25_PbI_2.7_Br_0.3_ | 1.14 | 23.4 | 75.6 | 20.2 | 0.1 | 2022 [S3] |
| 1.65 | Cs_0.17_FA_0.83_Pb(I_0.8_Br_0.2_)_3_ | 1.24 | 20.80 | 79.5 | 20.56 | 0.094 | 2022 [S4] |
|  |  | 1.21 | 20.55 | 74.0 | 18.4 | 1.00 |  |
| 1.71 | FA_0.9_Cs_0.1_Pb(I_0.7_Br_0.3_)_3_ | 1.27 | 20.32 | 81.22 | 20.84 | 0.09 | 2022 [S5] |
| 1.68 | (FA_0.65_MA_0.2_Cs_0.15_)Pb(I_0.8_Br_0.2_)_3_ | 1.16 | 20.94 | 81.8 | 19.92 | 0.07 | 2023 [S6] |
| 1.68 | Cs_0.22_(FA_0.8_MA_0.2_)_0.78_Pb(I_0.83_Br_0.14_Cl_0.03_)_3_ | 1.21 | 21.3 | 79.6 | 20.9 | 0.1 | 2024 [S7] |
| 1.67 | FA_0.7_Cs_0.25_MA_0.05_Pb(I_0.8_Br_0.2_)_3_ | 1.25 | 21.05 | 84.34 | 22.06 | 0.07 | 2024 [S8] |
|  |  | 1.26 | 20.15 | 76.98 | 19.63 | 1.02 |  |
| 1.67 | Cs_0.22_FA_0.63_MA_0.15_PbI_0.83_Br_0.14_Cl_0.03_ | 1.23 | 21.4 | 85.0 | 22.4 | 0.057 | 2024 [S9] |
|  |  | 4.78 | 20.8 | 79 | 19.6 | 9.8 |  |
| 1.66 | (Cs_0.22_FA_0.63_MA_0.15_)Pb(I_0.83_Br_0.14_Cl_0.03_)_3_ | 1.24 | 23.1 | 82.9 | 22.6 | 0.1 | 2024 [S10] |
| 1.68 | Cs_0.25_FA_0.75_Pb(I_0.8_Br_0.2_)_3_ | 1.20 | 20.72 | 85.37 | 21.31 |  | 2024 [S11] |
| 1.68 | Cs_0.05_FA_0.80_MA_0.15_PbI_2.25_Br_0.75_ | 1.24 | 20.6 | 84 | 21.5 | 1.05 | 2024 [S12] |
| 1.69 | FA_0.8_Cs_0.2_PbI_2.25_Br_0.75_ | 1.26 | 21.75 | 83.94 | 23.0 | 0.093 | Our work,  2025 |
|  |  | 6.34 | 4.16 | 76.47 | 20.2 | 10.5 |  |

**Supplementary References**

1. B. Chen, Z.J. Yu, S. Manzoor, S. Wang, W. Weigand et al., Blade-coated perovskites on textured silicon for 26%-efficient monolithic perovskite/silicon tandem solar cells. Joule **4**(4), 850–864 (2020). <https://doi.org/10.1016/j.joule.2020.01.008>
2. G. Yang, Z. Ni, Z.J. Yu, B.W. Larson, Z. Yu et al., Defect engineering in wide-bandgap perovskites for efficient perovskite–silicon tandem solar cells. Nat. Photonics **16**(8), 588–594 (2022). <https://doi.org/10.1038/s41566-022-01033-8>
3. Y. Zheng, X. Xu, S. Liu, G. Xu, Z. Bi et al., Blade coating high-quality formamidinium–cesium lead halide perovskites with green solvent for efficient and stable solar cells. Sol. RRL **6**(12), 2200737 (2022). <https://doi.org/10.1002/solr.202200737>
4. G.S. Jang, Y. Kim, Y.Y. Kim, J.J. Yoo, G. Kim et al., Ambient air-processed wide-bandgap perovskite solar cells with well-controlled film morphology for four-terminal tandem application. Sol. RRL **6**(8), 2200252 (2022). <https://doi.org/10.1002/solr.202200252>
5. X. Zhou, H. Lai, T. Huang, C. Chen, Z. Xu et al., Suppressing nonradiative losses in wide-band-gap perovskites affords efficient and printable all-perovskite tandem solar cells with a metal-free charge recombination layer. ACS Energy Lett. **8**(1), 502–512 (2023). <https://doi.org/10.1021/acsenergylett.2c02156>
6. Z. Zhang, J. Shang, H. Ge, Y. Zhang, Q. Chen et al., Suppressing halide phase segregation in wide-bandgap perovskite film by co-doping strategy for high-performance and stable perovskite solar cells. Mater. Today Phys. **37**, 101187 (2023). <https://doi.org/10.1016/j.mtphys.2023.101187>
7. A.S. Subbiah, L.V. Torres Merino, A.R. Pininti, V. Hnapovskyi, S. Mannar et al., Enhancing the performance of blade-coated perovskite/silicon tandems *via* molecular doping and interfacial energy alignment. ACS Energy Lett. **9**(2), 727–731 (2024). <https://doi.org/10.1021/acsenergylett.4c00070>
8. P. Jia, G. Chen, G. Li, J. Liang, H. Guan et al., Intermediate phase suppression with long chain diammonium alkane for high performance wide-bandgap and tandem perovskite solar cells. Adv. Mater. **36**(25), 2400105 (2024). <https://doi.org/10.1002/adma.202400105>
9. A.R. Pininti, A.S. Subbiah, C. Deger, I. Yavuz, A. Prasetio et al., Resolving scaling issues in self-assembled monolayer-based perovskite solar modules *via* additive engineering. Adv. Energy Mater. **15**(7), 2403530 (2025). <https://doi.org/10.1002/aenm.202403530>
10. A.S. Subbiah, S. Mannar, V. Hnapovskyi, A.R. Pininti, B. Vishal et al., Efficient blade-coated perovskite/silicon tandems *via* interface engineering. Joule **9**(1), 101767 (2025). <https://doi.org/10.1016/j.joule.2024.09.014>
11. X. Ge, Z. Huang, B. Shi, P. Wang, Z. Liu et al., Crystallization control of blade-coated wide bandgap FACs-based perovskite. Adv. Funct. Mater. **35**(12), 2417493 (2025). <https://doi.org/10.1002/adfm.202417493>
12. C. Duan, H. Gao, K. Xiao, V. Yeddu, B. Wang et al., Scalable fabrication of wide-bandgap perovskites using green solvents for tandem solar cells. Nat. Energy **10**(3), 318–328 (2024). <https://doi.org/10.1038/s41560-024-01672-x>
